# A highly sensitive Au@Pd NRs SERS microarray chip based on electrophoretic deposition for detection of GSH in the serum of colorectal cancer patients

**DOI:** 10.1039/d6ra01029f

**Published:** 2026-07-02

**Authors:** Chenxi Xie, Dong Zhang, Yijiang Wu, Yuan He, Yiman Ge, Bin Deng

**Affiliations:** a The First School of Clinical Medicine, Faculty of Medicine, Yangzhou University Yangzhou 225009 PR China xcx128914@qq.com 2937895362@qq.com 213780391@qq.com 3414085641@qq.com; b Department of Gastroenterology, Northern Jiangsu People's Hospital Yangzhou Jiangsu 225009 PR China; c Department of General Surgery, Guanyun County People's Hospital Lianyungang Jiangsu 222200 PR China zhangd123456@sina.com; d Northern Jiangsu People's Hospital Affiliated to Yangzhou University Yangzhou 225009 PR China jsyzdys@163.com

## Abstract

In this study, a highly sensitive surface-enhanced Raman scattering (SERS) microarray chip was developed for the detection of reduced glutathione (GSH) in the serum of colorectal cancer (CRC) patients. Gold-palladium nanorods (Au@Pd NRs) with peroxidase-like activity were synthesized and assembled into holes on the polydimethylsiloxane (PDMS)-indium tin oxide (ITO) glass substrate *via* electrophoretic deposition (EPD). The Au@Pd NRs catalyze the oxidation of 3,3′,5,5′-tetramethylbenzidine (TMB) to oxidized TMB (oxTMB) in the presence of hydrogen peroxide (H_2_O_2_), producing a characteristic SERS peak at 1605 cm^−1^. Prior to detection, a thiolated GSH aptamer was immobilized on the microarray chip to specifically capture GSH, effectively avoiding interference from other reducing substances in the complex serum matrix. The captured GSH then reduces oxTMB back to TMB, causing signal attenuation proportional to GSH concentration. The limit of detection (LOD) reached 93 pmol L^−1^, with a relative standard deviation (RSD) of 6.36% for reproducibility. Clinical serum analysis of 30 healthy individuals and 30 CRC patients demonstrated results consistent with the gold standard testing method of high-performance liquid chromatography (HPLC) (relative error < 8%), confirming the potential of this approach for the effective detection of GSH and early CRC diagnosis.

## Abstract 2

In this study, a highly sensitive surface-enhanced Raman scattering (SERS) microarray chip was developed for the detection of reduced glutathione (GSH) in the serum of colorectal cancer (CRC) patients.

## Graphical abstract

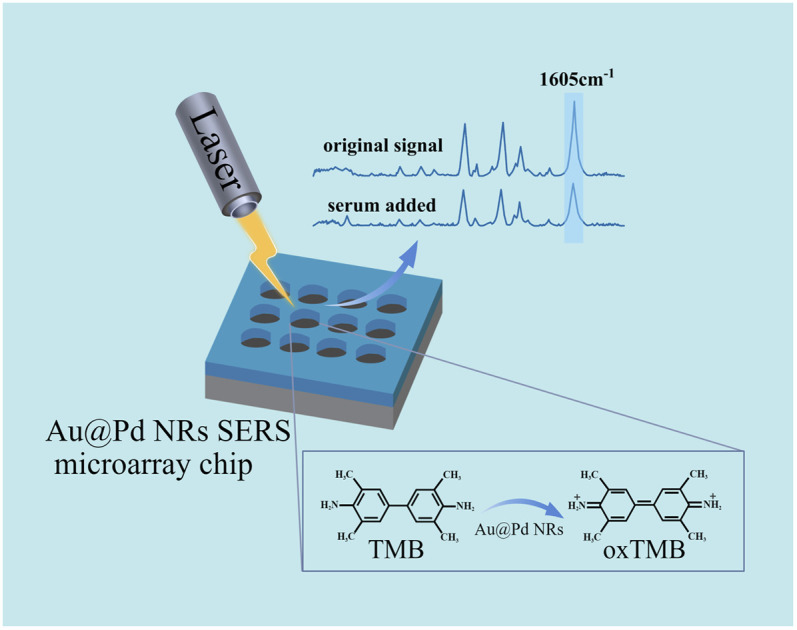

## 1. Introduction

Colorectal cancer (CRC) ranks as the third most prevalent malignant tumour globally and the second leading cause of cancer mortality, with nearly two million new cases diagnosed each year and approximately one million deaths annually.^1,2^ The 5-year survival rate for early-stage CRC patients exceeds 90%, but significant symptoms only appear in the advanced stages.^3,4^ Therefore, early screening for CRC is a crucial determinant in preventing metastasis, reducing mortality rates, improving prognosis, and enhancing future quality of life.^5^ Currently, colonoscopic biopsy is still considered the gold standard for clinically diagnosing CRC.^6,7^ However, it carries inherent limitations including high invasiveness, relatively low accuracy and sensitivity, and high cost.^8,9^ Consequently, developing an efficient, non-invasive, and highly sensitive detection method has become a priority in CRC screening.

Glutathione (GSH) is a small peptide composed of three amino acid residues and serves as the core molecule in intracellular thiol metabolism.^10^ This biomarker, which is crucial for the body's redox homeostasis, significantly influences the initiation and progression of CRC by scavenging reactive oxygen species,^11^ regulating inflammation signals,^12^ and maintaining DNA repair mechanisms.^13^ It is highly associated with CRC risk.^14^ Wang *et al.* identifies GSH as a key determinant of CRC cell survival, showing that enhanced GSH synthesis confers resistance to ferroptosis and supports tumor progression.^15^ Zhou *et al.* demonstrated that m^6^A-mediated upregulation of RRM2B enhances GSH synthesis, which promotes mitochondrial fusion and supports CRC progression.^16^ Currently, the detection methods for GSH mainly include the 5,5′-dithiobis(2-nitrobenzoic acid) (DTNB) colorimetric method and high-performance liquid chromatography (HPLC).^17,18^ Among them, HPLC is recognized as the analytical gold standard for GSH quantification due to its superior accuracy. While the DTNB method offers convenience for rapid assessment, it remains susceptible to interference from co-existing thiols, and HPLC, despite its precision, is constrained by complex pretreatment and bulky instrumentation.^19,20^ Therefore, developing a convenient and ultra-sensitive method for GSH detection is of great significance for the effective identification of CRC and meeting the needs for early diagnosis in large populations.

Surface-enhanced Raman scattering (SERS) is an optical sensing technology based on molecular vibrations that can quickly identify information such as the structure, molecular composition, and conformation of substances.^21,22^ Due to its high sensitivity, strong specificity, robust analytical capability, and non-invasive detection features,^23–25^ it shows great application potential in fields such as catalysis,^26^ environmental monitoring,^27^ food safety,^28^ biotechnology,^29,30^ and surface science.^31^ The enhancement property of metallic nanoscale substrate is the key to achieving highly sensitive SERS detection.^32^ Gold-palladium nanorods (Au@Pd NRs), as a novel type of nanoscale material, exhibit excellent SERS enhancement effects due to their regular shape, high aspect ratio, superior stability, and the favorable nanoscale gaps between adjacent particles.^33,34^ As a bimetallic material, Au and Pd work synergistically, resulting in catalytic performance that far exceeds that of either material alone.^35^ At the same time, the porous structure and rich electronic properties of the Pd shell confer excellent peroxidase catalytic activity, making it a unique bifunctional material with outstanding SERS performance and nanoenzyme catalytic capability, which shows great application potential.

**Fig. 1:**
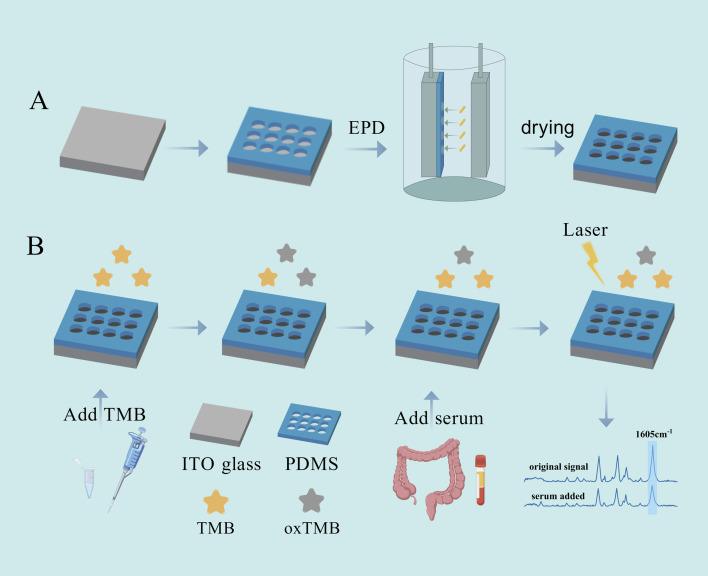
(A) Preparation of the SERS microarray chip, (B) SERS measurement and analysis. Created with https://biogdp.com/.^36^

Herein, this study successfully fabricated Au@Pd NRs arrays on a prepared substrate using electrophoretic deposition (EPD) technology, developing a SERS microarray chip with nanocatalytic functionality. First of all, the seed-mediated method was used to prepare Au NRs, and a layer of Pd shell was coated on the surface to form Au@Pd NRs. Next, a rectangular polydimethylsiloxane (PDMS) mold was fabricated by soft lithography and perforations were made on it, which was then adhered onto the cut indium tin oxide (ITO) glass surface using O_2_ plasma activation method to form a complete substrate for electrophoresis. Place the substrate in the Au@Pd NRs solution as the anode with an electric current applied. Due to the electric field force, the nanorods were adsorbed into the holes and the microarray chip were formed after drying. The hotspots generated by the aggregation of Au@Pd NRs can amplify SERS signals, enabling ultra-sensitive detection of trace biomarkers. At the same time, it can significantly enhance the activity of peroxidase, making it a catalyst for the reaction between hydrogen peroxide (H_2_O_2_) and 3,3′,5,5′-Tetramethylbenzidine (TMB). After TMB is oxidized, oxidized TMB (oxTMB) with strong SERS signals is produced. Specifically, to overcome the inherent cross-reactivity of general redox-based assays, a thiolated GSH aptamer was introduced to functionalize the Au@Pd NRs. Through specific spatial conformational matching and hydrogen bonding, the aptamer precisely captures GSH from the complex clinical serum of CRC patients. Subsequently, the captured GSH reduces oxTMB back to TMB, resulting in a decrease in oxTMB quantity and a weakening of its SERS signal. By quantifying the degree of SERS signal attenuation, this study successfully measured the levels of GSH in the serum of CRC patients and verified the results using the HPLC. The detection results of both methods are highly consistent. Compared to traditional approaches, this study integrates the efficient catalytic properties of nanomaterials with SERS detection technology to construct a SERS microarray chip. This microarray chip demonstrates higher sensitivity and specificity in detecting CRC-related biomarker GSH, providing a new avenue for the early diagnosis of CRC (Fig. 1).

## 2. Experimental section

### 2.1 Materials and reagents

Cetyltrimethylammonium bromide (CTAB), sodium borohydride (NaBH_4_), silver nitrate (AgNO_3_), and anhydrous ethanol were procured from Sinopharm Chemical Reagent Co., Ltd TMB, H_2_O_2_, 4-mercaptobenzoic acid (4-MBA), chloroauric acid (HAuCl_4_), chloropalladateII (H_2_PdCl_4_), ascorbic acid (AA), homocysteine (Hcy), cysteine (Cys), PDMS, crosslinking agent, *N*-acetylcysteine (NAC), formic acid and ethylenediaminetetraacetic acid (EDTA) were purchased from Shanghai Aladdin Co.,Ltd. Glutathione (GSH), GSH aptamer (5′-SH-C6-AAACGGGAGGAGCATATGCTCTGGTAAG-3′) was purchased from Shanghai Sango Biotechnology Co., Ltd. ITO glass was purchased from Zhuhai Kaivo Optoelectronic Technology Co., Ltd. Trichloroacetic acid (TCA) and acetonitrile were purchased from Sinopharm Chemical Reagent Co., Ltd. Deionised water with a resistivity of 18.2 MΩ was used throughout the experiments.

### 2.2 Equipment

Instruments employed included a field emission scanning electron microscope (FESEM, S-4800II, Hitachi, Japan), transmission electron microscope (TEM, Tecnai 12, Philips, Netherlands), field emission transmission electron microscopy (Tecnai G2F30S-TWIN, FEI), ultraviolet-visible spectrophotometry (Cary 60, Agilent), DXRxi micro-Raman spectroscopy (ThermoFisher, USA) and HPLC (Agilent 1260 Infinity II, Agilent Technologies, USA, equipped with a C18 chromatographic column and a UV-vis detector).

### 2.3 Synthesis of Au@Pd NRs

First, mix 19.5 mL of CTAB solution (0.1 M), 0.3 mL of HAuCl_4_ solution (0.01 M) evenly, then add 1.8 mL of freshly prepared ice-cold NaBH_4_ solution (0.01 M). Stir at low speed for 120 seconds until the color becomes light brown, then let it stand for 30 minutes to form Au seed solution. Next, prepare 100 mL of CTAB solution (1.0 M) and add 2 mL of AgNO_3_ solution (0.01 M) to it. After letting this mixture stand for 20 minutes, add 100 mL of HAuCl_4_ solution (0.01 M) and stir vigorously for 20 minutes. Add 0.5 mL of AA (0.1 M) and Au seed solution, continue stirring for 30 seconds, then let it stand at room temperature for 12 hours to obtain the Au NR solution. Take 10 mL of the synthesized Au NR solution and, under low-speed stirring conditions, add 0.1 mL of 0.1 M H_2_PdCl_4_ and 6 mL of freshly prepared 0.1 M AA. Then let it stand at room temperature for another 8 hours to obtain the Au@Pd NRs solution.

### 2.4 Preparation of SERS microarray chip

In the beginning, AutoCAD software was used to design the layout of the photolithography template, followed by the printing. PDMS was mixed with the crosslinking agent (10 : 1 ratio), poured into the prepared mold and cured at 70 °C for 3 hours. Next, cool it to room temperature and manually drill 4 × 3 circular holes (with a diameter of 3 mm) on the surface. Once the ultrasonic cleaning was performed, treat the PDMS cuboid with the corresponding size of ITO glass using an oxygen plasma for 5 minutes and bond them together. Place them in a 70 °C oven and heat for 2 hours. After cooling it to room temperature, a simple substrate was formed.

In this process, the prepared materials below and the Au@Pd NRs solution were used for the electric field deposition experiment. Place the ITO glass with PDMS attached and the original ITO glass into the Au@Pd NRs solution as the anode and cathode respectively. Then, apply a constant electric field using a potentiostat with a voltage of 5 V cm^−1^ and a duration of 10 minutes. After drying with N_2_ stream, it can be seen that the Au@Pd NRs are deposited in the holes to form a black disc-like structure. In this way, a SERS microarray chip was completely fabricated.

### 2.5 Collection and processing of clinical samples

The serum samples were obtained from Northern Jiangsu People's Hospital. All experiments were conducted in accordance with relevant national laws and institutional guidelines, specifically the “Measures for the Ethical Review of Life Science and Medical Research Involving Humans (2023)” of China and international ICH-GCP guidelines, as well as the principles of the Declaration of Helsinki. Ethical approval was granted by the Medical Ethics Committee of Northern Jiangsu People's Hospital (approval number: 2024ky308; approval date: 14th November 2024). Written informed consent was obtained from all participants prior to sample collection. The study enrolled 30 healthy volunteers and 30 patients diagnosed with CRC, aged between 40 and 80 years old. All serum samples were stored at −80 °C until analysis.

### 2.6 SERS measurement

The study standardized the concentration of serum samples from healthy individuals to 10^−5^ mol L^−1^ by preprocessing and exogenous addition of GSH. Subsequently, a gradient dilution strategy was employed to construct a series of GSH standard solutions with concentrations ranging from 10^−10^ to 10^−4^ mol L^−1^. Incubate the SERS microarray chip with the GSH aptamer solution (50 µM) at room temperature for 2 hours to allow the formation of stable Au–S bonds. Then, a mixture solution of 1 mM TMB and 0.8 M H_2_O_2_ (controlled at pH 4.5) was dripped onto the array, followed by a constant temperature reaction at 35 °C for twenty minutes. When the solution exhibited a clear blue color, Raman analysis was performed on the SERS detection platform with the following settings: laser wavelength of 785 nm, laser power of 5 mW, exposure time of 10 seconds with 3 accumulations, objective lens magnification of 50× and grating of 1200 lines per mm.

### 2.7 Validation of GSH detection by HPLC

To validate the accuracy of the SERS microarray chip, the GSH concentration in clinical serum samples was simultaneously determined using the HPLC method as the gold standard. Briefly, serum samples were deproteinized with 10% (w/v) TCA and centrifuged at 12 000 rpm for 10 min. To maintain the stability of GSH and prevent oxidation during sample preparation, 1 mM EDTA was added to the serum samples. The supernatant was filtered and injected into an HPLC system equipped with a C18 column (4.6 mm × 250 mm, 5 µm). The mobile phase consisted of a mixture of 0.1% formic acid in water and acetonitrile (95 : 5, v/v). The flow rate was set at 1.0 mL min^−1^, the column temperature was maintained at 30 °C, and the injection volume was 20 µL. The detection wavelength was set at 215 nm. The GSH concentrations in clinical samples were quantified based on a standard calibration curve, and the results were compared with those obtained from our SERS microarray chip.

## 3. Results and discussion

### 3.1 Characterisation of Au@Pd NRs

**Fig. 2:**
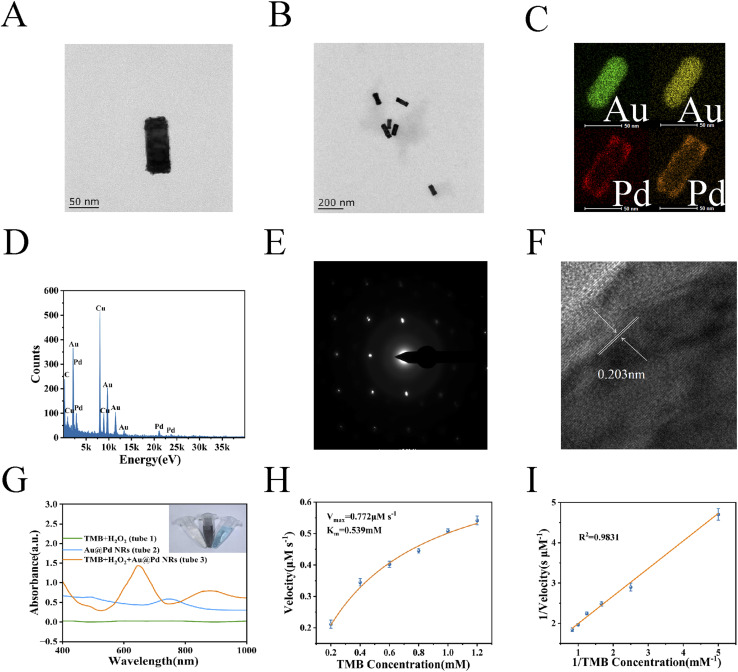
(A) and (B) TEM images of Au@Pd NRs, (C) elemental mapping image of Au@Pd NRs, (D) energy-dispersive spectroscopy spectrum of Au@Pd NRs, (E) selected area electron diffraction image of Au@Pd NRs, (F) HRTEM image of Au@Pd NRs, (G) UV-visible absorption spectra of three different cases and solution colour, (H) Michaelis–Menten curve of Au@Pd NRs, (I) double-reciprocal plot of Au@Pd NRs.

The morphology and size of Au@Pd NRs were observed using TEM, as shown in Fig. 2(A). The individual Au@Pd NRs exhibited a rod-like shape with a length of approximately 85 nm, a diameter of about 30 nm, and an aspect ratio of around 2.8 : 1. Fig. 2(B) indicates that the Au@Pd NRs have a uniform morphology, consistent size, and good dispersion. Fig. 2(C) and (D) collectively demonstrate that Au and Pd are the main constituents of Au@Pd NRs, while Fig. 2(C) further visualizes the coating relationship between Pd and Au through elemental mapping. In the lattice diffraction pattern of Fig. 2(E), the spots are arranged in a regular array, indicating that the sample has a single crystal structure. The HRTEM image in Fig. 2(F) indicates that the interplanar spacing is 0.203 nm. Fig. 2(I) indicates that the solution is colorless when TMB and H_2_O_2_ are present simultaneously. After the addition of Au@Pd NRs, the solution quickly turned blue, and a strong absorption peak appeared at 644 nm, indicating that the nanozyme catalyzes the oxidation of TMB to produce oxTMB. Fig. 2(F) and (G) measured the activity of the nanozyme using Michaelis–Menten curves and their corresponding double-reciprocal plots, calculating that the *V*_max_ of Au@Pd NRs is 0.772 µM s^−1^ and *K*_m_ is 0.539 mM.

### 3.2 Characterisation of the arrayed hydrophobic substrate

**Fig. 3:**
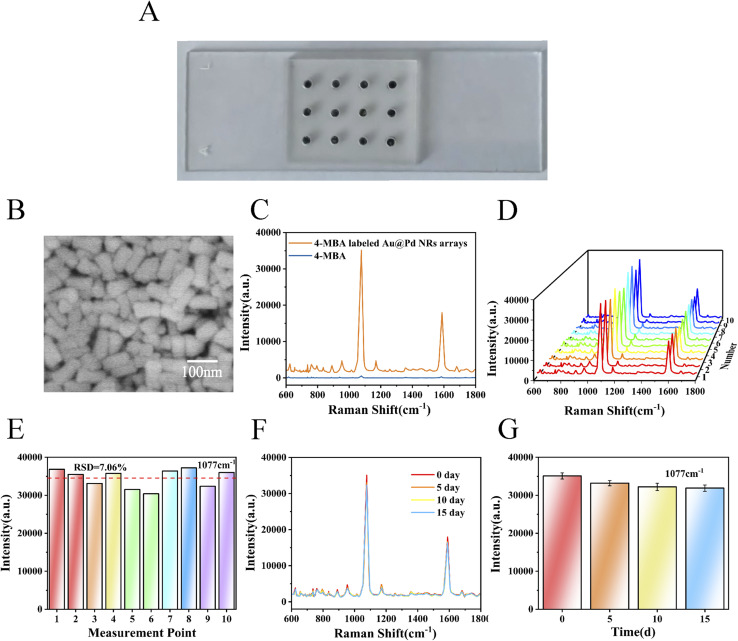
(A) Sample image of the sediments of Au@Pd NRs left by electrostatic self-assembly, (B) SEM image of the array, (C) SERS spectra of pure 4-MBA (10^−2^ mol L^−1^) and 4-MBA (10^−9^ mol L^−1^) labelled Au@Pd NRs array, (D) SERS spectra from ten randomly selected points on the surface of 4-MBA (10^−9^ mol L^−1^) labelled Au@Pd NRs array, (E) signal intensity histograms at 1077 cm^−1^ for different positions, (F) SERS spectra of the 4-MBA (10^−9^ mol L^−1^) labelled Au@Pd NRs array after storage for different durations (0, 5, 10, 15 days), (G) signal intensity histograms at 1077 cm^−1^ for different storage time (0, 5, 10, 15 days).

Fig. 3(A) shows the image of the Au@Pd NRs array of the chip after EPD. The array is fabricated using the self-assembly method based on electric field force. The closely arranged Au@Pd NRs form abundant nanogaps, and the uniform nanogaps can significantly enhance the local electromagnetic field, generating numerous hotspots, thereby enhancing the sensitivity of detection.^37^Fig. 3(B) is the SEM image of the array. As can be seen from the figure, Au@Pd NRs are closely arranged and densely stacked, forming abundant hotspots. Fig. 3(C) presents a comparative experiment using 4-MBA as the probe molecule, verifying the SERS enhancement performance of the SERS microarray chip compared to that of a simple substrate. Compared to pure 4-MBA (10^−2^ mol L^−1^), the 4-MBA labelled Au@Pd NRs (10^−9^ mol L^−1^) exhibited significantly enhanced characteristic Raman signals. The enhanced factor (EF) calculated based on this reached 5.1 × 10^8^, confirming that Au@Pd NRs possess exceptional SERS activity. Additionally, Fig. 3(D) and (E) show the SERS spectra of ten randomly selected locations on the surface of 4-MBA (10^−9^ mol L^−1^) labelled Au@Pd NRs array along with their signal intensity at 1077 cm^−1^. It can be observed that their waveforms are relatively consistent, with minor differences in characteristic peak intensity. The RSD is only 7.06%, indicating good uniformity. Fig. 3(F) and (G) show the SERS spectra of the 4-MBA (10^−9^ mol L^−1^) labelled Au@Pd NRs array after being placed for 0, 5, 10, and 15 days, along with the signal intensity at 1077 cm^−1^. The spectral lines are essentially overlapping, with a slight decrease in intensity. After being stored at room temperature for 15 days, the SERS spectral intensity at 1077 cm^−1^ only decreased to 91.48%, demonstrating that the 4-MBA labelled microarray chip has good stability.

### 3.3 Optimisation of experimental parameters

**Fig. 4:**
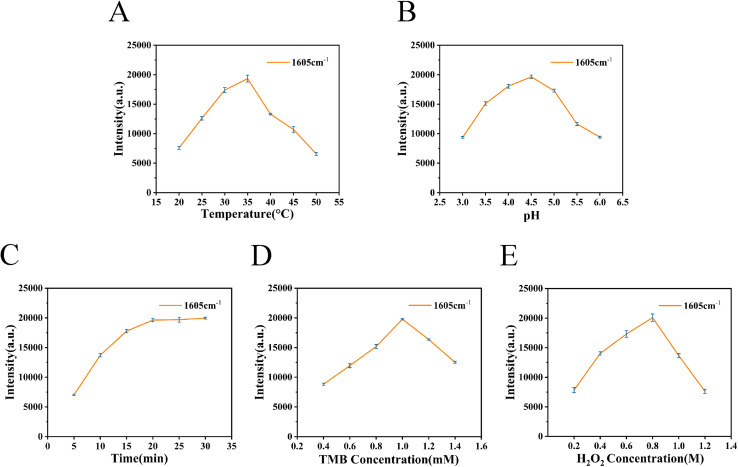
(A) Signal intensity at 1605 cm^−1^ at different temperatures, (B) signal intensity at 1605 cm^−1^ at different pH values, (C) signal intensity at 1605 cm^−1^ at different incubation times, (D) signal intensity corresponding to different TMB concentrations at a fixed H_2_O_2_ concentration, (E) signal intensity corresponding to different H_2_O_2_ concentrations at a fixed TMB concentration.

The SERS microarray chip has been prepared for further study. To achieve the optimal experimental results, a systematic optimization of the test parameters was conducted. It can be seen from Fig. 4(A) and (B) that excessively high or low temperatures and pH levels significantly affect the nanoenzyme activity of Au@Pd NRs, causing the signal intensity at 1605 cm^−1^ to exhibit an initial increase followed by a decrease. Fig. 4(C) shows that after the reaction has lasted for 20 minutes, the signal at 1605 cm^−1^ gradually stabilizes. Therefore, optimizing the reaction time to 20 minutes is beneficial in maximizing detection efficiency. Fig. 4(D) indicates that when the TMB concentration exceeds 1 mM, the signal intensity decreases rapidly. This may be due to the fact that excess TMB occupied the active sites on the surface of Au@Pd NRs, hindering H_2_O_2_ adsorption and O–O bond cleavage, leading to a phenomenon known as “substrate inhibition”.^38^Fig. 4(E) similarly exhibits an initial increase followed by a decrease, which may be due to the fact that when the concentration of H_2_O_2_ exceeds 0.8 M, high concentrations of H_2_O_2_ oxidize Pd(0) active sites into Pd(ii), leading to a sharp decrease in the number of catalytic sites and a decline in apparent rate. In summary, when the reaction system temperature is 37 °C, pH is 4.5, incubation time is 20 minutes, TMB concentration is 1 mM, and H_2_O_2_ concentration is 0.8 M, Au@Pd NRs exhibit the strongest peroxidase-like activity.

### 3.4 Functional validation

**Fig. 5:**
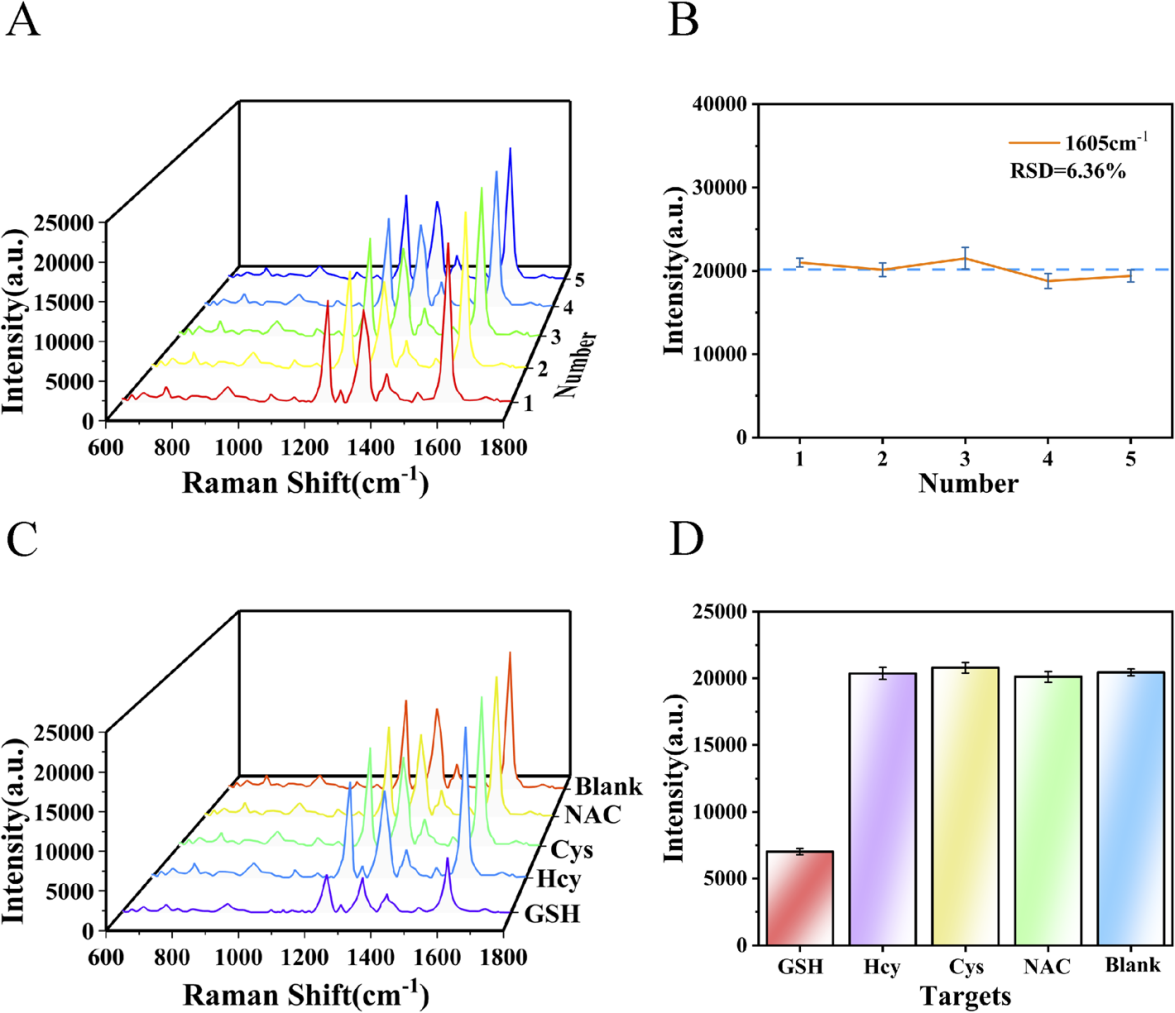
(A) SERS spectra of microarray chips from five different batches, (B) line graph of signal intensity at 1605 cm^−1^ for different batches, (C) SERS spectra for detecting different targets (GSH, Hcy, Cys, NAC and blank), (D) histogram of SERS signal intensity at 1605 cm^−1^ for different targets.

Under optimized experimental conditions, this study systematically evaluated the reproducibility and specificity of the constructed microarray chips. Fig. 5(A) and (B) show the Raman spectra of five independently prepared arrays in the detection area, along with the SERS intensity at the characteristic peak of 1605 cm^−1^. Both their shape and intensity are highly consistent, with the relative standard deviation (RSD) of 6.36% for the peak intensities, indicating good reproducibility in the chip preparation process. In addition, this study introduces Hcy, Cys, and NAC as interfering substances for testing. The results are shown in Fig. 5(C) and (D). The signal intensity at 1605 cm^−1^ significantly decreases only in the presence of the target detection substance GSH, while all interference groups exhibit high-intensity Raman signals similar to the blank group. This indicates that this chip reacts specifically with GSH. In summary, this microarray chip demonstrates excellent reproducibility and specificity. This highly specific response is fundamentally attributed to the introduction of the GSH aptamer. The aptamer precisely recognizes and captures target GSH through spatial conformational matching and multi-site non-covalent interactions (such as hydrogen bonding and electrostatic forces). This biological recognition mechanism effectively shields the catalytic system from the competitive reduction by co-existing analogues like Hcy, Cys, and NAC. In summary, this microarray chip demonstrates excellent reproducibility and exceptional specificity.

### 3.5 Quantitative characterisation for GSH detection

**Fig. 6:**
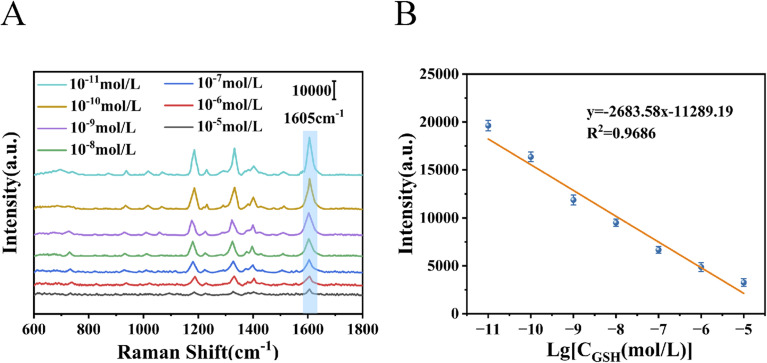
(A) SERS spectra corresponding to different GSH concentrations in serum, (B) fitted curve of signal intensity at 1605 cm^−1^*versus* logarithm of GSH concentration.

To evaluate the sensitivity of the SERS microarray chip, serum samples containing different concentrations of GSH (10^−11^ to 10^−5^ mol L^−1^) were spotted onto the surface of the microarray chip. The characteristic Raman peak at 1605 cm^−1^ is assigned to the vibrational coupling between aromatic ring C=C stretching and imine C=N stretching modes in the diimine-structured oxTMB, reflecting the extended π-conjugation upon oxidation.^39,40^ As shown in Fig. 6(A), as the concentration of GSH continues to increase, more oxTMB is reduced, and there is a significant decreasing trend in the SERS signal at the characteristic peak. As shown in Fig. 6(B), the signal intensity of oxTMB at 1605 cm^−1^ shows a linear correlation with the logarithm of GSH concentration, and its linear regression equation is *y* = −2683.58*x* − 11289.19, with a correlation coefficient (*R*^2^) of 0.9686. The limit of detection (LOD) was calculated using the formula LOD = 3SD/*K* (where SD is the standard deviation of the SERS intensity at 1605 cm^−1^ for blank samples, and *K* is the slope of the fitted curve), resulting in a low LOD of up to 93 pmol L^−1^. Compared with previous detection methods, this approach has higher sensitivity (Table 1) and can be used for the precise detection of clinical CRC serum samples.

**Table 1:** Comparison of the present method with previously reported GSH detection methods

Strategy	Linear range	LOD	References
Fluorescence	2.5–1000 nmol L^−1^	1.54 nmol L^−1^	41
HPLC	0.05–1.5 mmol L^−1^	0.50 µmol L^−1^	18
Colorimetric	1–300 µmol L^−1^	5 nmol L^−1^	42
Electrochemical	0.1–11 µmol L^−1^	0.05 µmol L^−1^	43
SERS	10^−5^–10 µmol L^−1^	93 pmol L^−1^	This work

### 3.6 Analysis of clinical serum samples

**Fig. 7:**
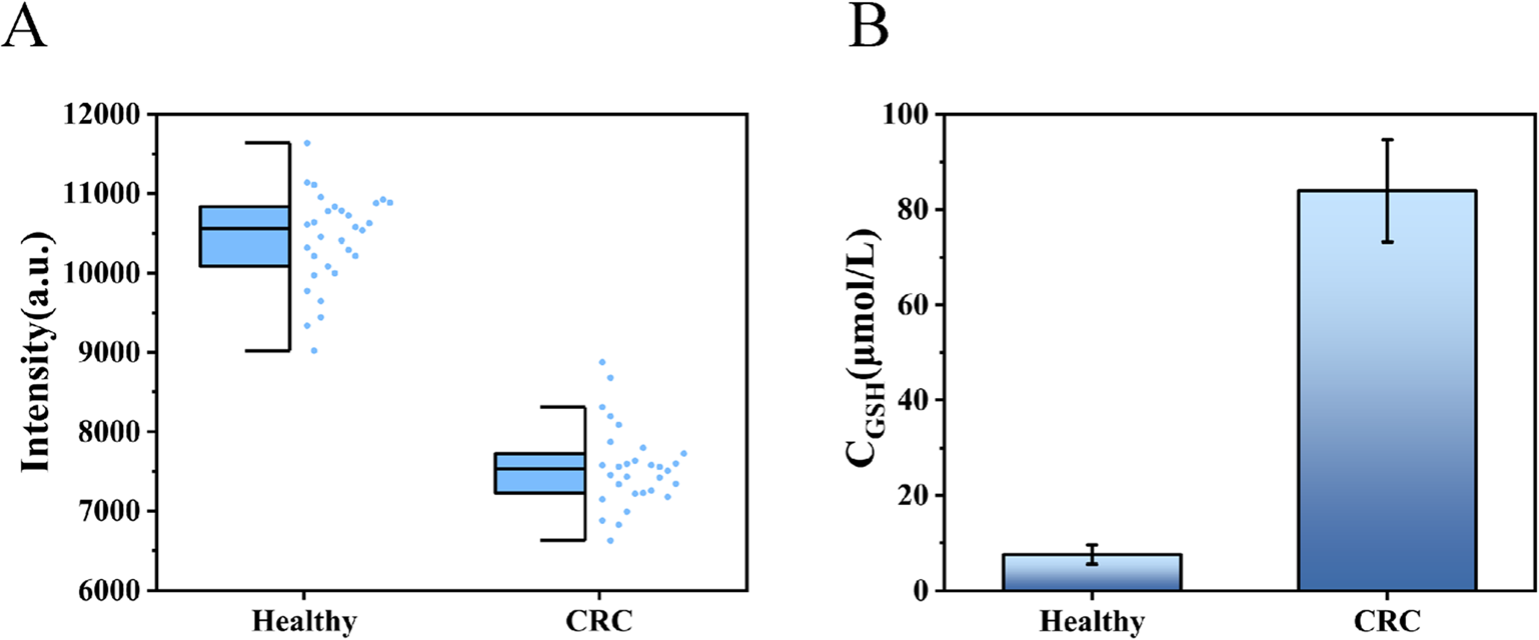
(A) Half-box line plot of SERS intensity at 1605 cm^−1^ from serum of healthy individuals and CRC patients, (B) histograms of GSH concentrations in serum from healthy individuals and CRC patients.

As shown in Fig. S1 in the SI, we collected clinical samples and serum samples from 30 healthy individuals and 30 CRC patients. To further verify the applicability of the SERS microarray chip in clinical applications, this study conducted SERS measurement of GSH in the collected serum samples. Fig. 7(A) shows the average SERS intensity at 1605 cm^−1^ corresponding to GSH in the serum samples of these participants. Fig. 7(B) shows the GSH concentration obtained by substituting the signal intensity of two sample groups at 1605 cm^−1^ into a fitting equation. As is shown in Table 2, the RSD between SERS and HPLC for GSH in healthy individuals were −3.77%, and in CRC patients were −7.30%, demonstrating that this SERS microarray chip possesses high accuracy in clinical serum sample analysis.

**Table 2:** Expression levels of GSH in serum samples detected by SERS and HPLC

Sample	SERS (µmol L^−1^)	HPLC (µmol L^−1^)	Relative error (%)
Healthy people	7.43 ± 1.78	7.15 ± 1.70	−3.77
CRC patients	83.93 ± 10.71	77.80 ± 10.54	−7.30

## 4. Conclusions

This study successfully designed a novel aptamer-functionalized SERS microarray chip that enables highly sensitive detection of GSH in clinical serum samples. The Au@Pd NRs synthesized by this method exhibited good peroxidase-like activity and SERS enhancement capabilities. The nanogaps between the closely arranged Au@Pd NRs assembled on the substrate generated a large number of hotspots, further amplifying the SERS signal and achieving a low LOD of 93 pmol L^−1^. At the same time, it has been proven that this chip possesses superior specificity, stability, reproducibility, and accuracy. Compared with existing methods, this study provides a simple, rapid, highly sensitive, and non-invasive solution for the detection of GSH in clinical samples, which offers new technological means for the early diagnosis of CRC and has significant potential for clinical application.

## Conflicts of interest

There are no conflicts to declare.

## Supplementary Material

RA-OLF-D6RA01029F-s001

## Data Availability

All data generated or analysed during this study are included in this article. Supplementary information (SI) is available. See DOI: https://doi.org/10.1039/d6ra01029f.
