# Supplementary material for: A highly sensitive Au@Pd NRs SERS microarray chip based on electrophoretic deposition for detection of GSH in the serum of colorectal cancer patients
Source: RSC Adv. 2026 Jul 2;16(34):31993–2002. doi: 10.1039/d6ra01029f (PMC13325425; doi:10.1039/d6ra01029f)
Supplement: RA-016-D6RA01029F-s001 [file RA-016-D6RA01029F-s001.pdf]

Supplementary Information (SI) for RSC Advances.
This journal is © The Royal Society of Chemistry 2026

Supplementary Information

A highly sensitive Au@Pd NRs SERS microarray chip based on electrophoretic deposition for detection of GSH in the serum of colorectal cancer patients

Chenxi Xie[1#], Dong Zhang[3#], Yijiang Wu[1], Yuan He[1], Yiman Ge[1], Bin Deng[2,4*]

[1]The First School of Clinical Medicine, Faculty of Medicine, Yangzhou University, Yangzhou, 225009, PR China.

[2]Department of Gastroenterology, Northern Jiangsu People's Hospital, Jiangsu, Yangzhou, 225009, PR China.

[3]Department of General Surgery, Guanyun County People's Hospital, Jiangsu, Lianyungang, 222200, PR China.

[4]Northern Jiangsu People's Hospital Affiliated to Yangzhou University, Yangzhou, 225009, PR China.

[#]Both authors contributed equally to this work.

*Correspondence: Bin Deng (jsyzdys@163.com)

Characterisation of clinical samples

All subjects enrolled were selected through imaging, colonoscopy, and pathological examinations. Fig.S1(A) shows the CT scan of a CRC patient. Compared to the scan of a healthy individual in Fig.S1(D), there is a noticeable irregular thickening of the intestinal wall and narrowing of the intestinal lumen. Fig.S1(B) is the colonoscopy image of a healthy individual , showing the normal structure of the intestinal mucosa. Fig.S1(E) shows that the patient with CRC developed exophytic lesions in the intestinal lumen, accompanied by local ulcers and bleeding. Fig.S1(C) and (F) show pathological sections of a healthy individual and a CRC patient, where abnormal hyperplasia of the glandular tubules is observed in the CRC patient.

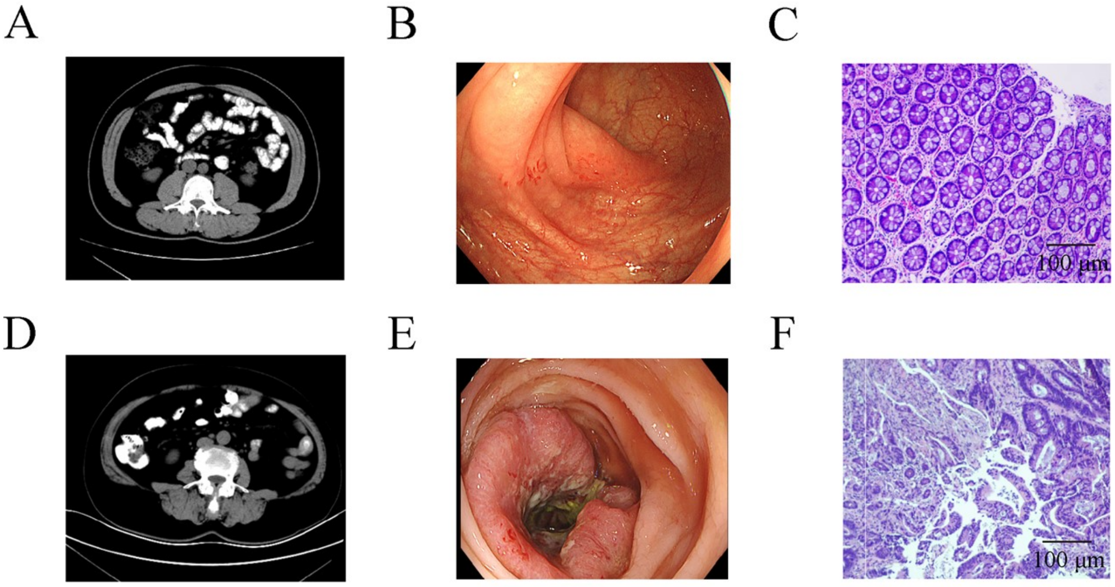

Fig.S1 (A) CT scan of a healthy individual, (B) Colonoscopic image of a healthy individual, (C) Histopathological section of a healthy individual, (D) CT scan of a CRC patient, (E) Colonoscopic image of a CRC patient, (F) Histopathological section of a CRC patient.
